# *Paracoccus denitrificans*: a genetically tractable model system for studying respiratory complex I

**DOI:** 10.1038/s41598-021-89575-9

**Published:** 2021-05-12

**Authors:** Owen D. Jarman, Olivier Biner, John J. Wright, Judy Hirst

**Affiliations:** The Medical Research Council Mitochondrial Biology Unit, University of Cambridge, The Keith Peters Building, Cambridge Biomedical Campus, Hills Road, Cambridge, CB2 0XY UK

**Keywords:** Bioenergetics, Enzymes, Oxidoreductases, Biochemistry, Membrane proteins, Metalloproteins, Mitochondrial proteins

## Abstract

Mitochondrial complex I (NADH:ubiquinone oxidoreductase) is a crucial metabolic enzyme that couples the free energy released from NADH oxidation and ubiquinone reduction to the translocation of four protons across the inner mitochondrial membrane, creating the proton motive force for ATP synthesis. The mechanism by which the energy is captured, and the mechanism and pathways of proton pumping, remain elusive despite recent advances in structural knowledge. Progress has been limited by a lack of model systems able to combine functional and structural analyses with targeted mutagenic interrogation throughout the entire complex. Here, we develop and present the α-proteobacterium *Paracoccus denitrificans* as a suitable bacterial model system for mitochondrial complex I. First, we develop a robust purification protocol to isolate highly active complex I by introducing a His_6_-tag on the Nqo5 subunit. Then, we optimize the reconstitution of the enzyme into liposomes, demonstrating its proton pumping activity. Finally, we develop a strain of *P. denitrificans* that is amenable to complex I mutagenesis and create a catalytically inactive variant of the enzyme. Our model provides new opportunities to disentangle the mechanism of complex I by combining mutagenesis in every subunit with established interrogative biophysical measurements on both the soluble and membrane bound enzymes.

## Introduction

Complex I (NADH:ubiquinone oxidoreductase) is a large, multisubunit, membrane-bound enzyme that couples the oxidation of NADH and reduction of ubiquinone to the translocation of four protons across the inner mitochondrial membrane, or cytoplasmic membrane in prokaryotes, contributing to the proton motive force (∆p) that is used to power ATP synthesis^1–3^. Complex I is also a key site for cellular reactive oxygen species generation^4,5^, particularly during ischemia reperfusion injury^6^. Due to its essential role in metabolism and energy production, mutations in complex I and its associated proteins are the origin of a wide range of neuromuscular and metabolic disorders^7,8^.

In its most minimal form, as typically found in α-proteobacteria, complex I is composed of 14 conserved catalytic ‘core’ subunits; the seven core subunits of the hydrophilic domain catalyze electron transfer from NADH to ubiquinone, and the seven core subunits in the membrane domain contain the proton pumps^9,10^. The mammalian enzyme contains an additional 31 ‘supernumerary’ subunits that have been accumulated during evolution and are required for assembly, stability or regulation of the complex^11,12^. Despite the central importance of complex I in oxidative phosphorylation, both the coupling mechanism that captures the energy released from the electron transfer reaction and consumes it to drive proton translocation, and the mechanism and pathways of the proton pumps, remain elusive.

In the last decade, the ‘resolution revolution’ has resulted in a surge of complex I structural data, especially resulting from cryoEM analyses, including native enzyme structures from bacteria, yeast, plants and mammals^10,11,13–16^, and substrate and inhibitor bound states^17–20^. These structures, along with molecular dynamic simulations^2,21,22^, have identified intriguing structural elements and key residues that are likely involved in the coupling mechanism and proton transfer pathways. However, probing the catalytic roles of these elements and sites further is hindered by lack of model systems that first, are suitable for both functional and structural analyses, and second, allow genetic manipulation of the core membrane domain subunits, which are encoded on the mitochondrial DNA in eukaryotes. Robust methods for creating site-directed mutants in the mitochondrial encoded proteins in eukaryotes are not yet available, rendering the core membrane domain subunits inaccessible to mutagenesis in model systems such as *Yarrowia lipolytica*, the popular yeast model system for complex I, as well as in mammalian species. Development of an experimentally versatile and fully genetically tractable bacterial model system thus has a crucial role to play in addressing mechanistic questions.

The α-proteobacterium *Paracoccus denitrificans* is a strong candidate for a bacterial model of the mitochondrial enzyme. *P. denitrificans* is a close relative of the protomitochondrion^23^ and its respiratory chain possesses similarities to the contemporary mitochondrial chain that are uncommon among other bacterial species^23,24^. First, *P. denitrificans* uses ubiquinone-10 as its sole quinone in the cell, the same as used by the human respiratory chain and in many mammalian species^23,25^. Second, *P. denitrificans* complex I shares much higher sequence similarity with the mammalian complexes, particularly in the hydrophilic domain, than the other bacterial models currently in use (*Escherichia coli* and *Thermus thermophilus*)^26^. Third, *P. denitrificans* complex I possesses three supernumerary subunits with mitochondrial homologues^26^, which are not present in other bacterial models. Finally, *P. denitrificans* is known to form respiratory supercomplexes, including complex I-containing respirasomes^27^, and its electron transport chain composition matches that of the canonical mitochondrial chain; it contains both complex III (cytochrome *bc*_1_) and complex IV (cytochrome *c* oxidase). These features have led to *P. denitrificans* being usefully employed in the past as a model system for studies of complex III and IV^28,29^. In combination, these observations suggest that *P. denitrificans* presents exciting opportunities to develop a bacterial model system for detailed and comprehensive studies of mitochondrial complex I.

To date, only *E. coli and T. thermophilus* have been used as bacterial model systems for studying complex I. While the first published complex I structure came from *T. thermophilus*, it has not been utilized widely beyond structural work, likely due to the difficulty of growing cells at 70 °C and the challenge of biophysical experiments at elevated temperatures^9^. In contrast, the *E. coli* model system has been heavily employed in the functional interrogation of complex I. Extensive mutagenesis studies in *E. coli* have helped identify essential residues in the complex, such as conserved glutamate and lysine residues that span the central axis of the membrane domain, likely involved in charge propagation^21,30–33^. However, much of this work was carried out without the benefits of the detailed structural information now available. In addition, despite its wide use, the *E. coli* system has some drawbacks that limit its relevance as a model for the mitochondrial enzyme, particularly when compared to *P. denitrificans*: it uses menaquinone or ubiquinone-8 as a terminal electron acceptor^25^, has a lower sequence homology than *P. denitrificans* to the mitochondrial enzyme^26^ and *E. coli* lacks complex III and a cytochrome *c* oxidase and thus cannot form respirasome-like supercomplexes^34^.

Perhaps the most appealing aspect of using *P. denitrificans* complex I to study the enzyme’s catalytic mechanism is that it is possible to form well-coupled energy-transducing vesicles from *P. denitrificans* membranes, a feat that has not yet proved possible for other bacterial or yeast model systems. *P. denitrificans* sub-bacterial particles (SBPs, inverted cytoplasmic membrane vesicles) catalyze NADH-driven ATP synthesis both rapidly and efficiently, and they have been utilized to determine the number of protons translocated across the membrane by complex I for each NADH oxidized (the proton stoichiometry)^35,36^. Attempts to apply the same approach to *E. coli* vesicles did not succeed because the vesicles were not sufficiently well coupled and the data did not converge to provide a robust stoichiometry value. The only published study of the stoichiometry of the *E. coli* enzyme was only able to report a value of at least three^37^. The lack of a robust method to determine the proton pumping stoichiometry of *E. coli* complex I compromises its use, as the stoichiometry is a key biophysical property that underpins mechanistic assessment of site-directed mutants.

Here, we present *P. denitrificans* as a genetically tractable model system that can be used to generate and study mutations in every core subunit of complex I. Despite being considered unstable when not associated with its supercomplex^27^, a four-column purification strategy for purifying intact complex I from *P. denitrificans* (*Pd*-CI) has been described previously^26^. Here, we describe a robust, streamlined two-step purification to isolate catalytically active *P. denitrificans* complex I using a genetically engineered affinity tag. Following characterization of the pure enzyme, we describe its reconstitution into liposomes to demonstrate that it remains coupled and retains proton pumping activity. Finally, we describe a *P. denitrificans* strain engineered to be amenable to the creation of deleterious complex I variants, produced by introducing an alternative NADH dehydrogenase as an artificial bypass for complex I essentiality. A point mutation in a conserved charge residue of the Nqo13 (ND4) subunit was then created and confirmed to possess no catalytic activity, demonstrating the versatility and capability of our system.

## Results

### Insertion of a His_6_-tag into the complex I operon

To develop a robust protocol for isolating complex I from *P. denitrificans* we introduced an affinity purification tag onto the C-terminus of the Nqo5 (NDUFS3) subunit. The affinity tag contained six histidine residues attached to the Nqo5 subunit by six alanine linker residues. The same tag, on the homologous subunit, has been used to purify complex I from the yeast model *Y. lipolytica*^17,38,39^*.* Here, we introduced an unmarked insertion of the His_6_-tag into the chromosomal DNA of *P. denitrificans* strain *Pd*1222-∆Hy^35^ by suicide vector-mediated homologous recombination, an approach previously used to create unmarked deletions in the *P. denitrificans* genome (see “Materials and methods”)^35,40,41^. The genetic modification was supported by expression of an alternative NADH dehydrogenase (NDH-2), as discussed in detail below. The correct and specific insertion of the purification tag on *nqo5* was confirmed by sequencing, and the sequences of the all known complex I subunits were checked in the final strain. We refer to this strain, which is formally termed *Pd*1222-∆Hy-Nqo5^His6^, as *Pd*-Nqo5^His6^. Expression of the tagged Nqo5 subunit was subsequently confirmed by Western blot analyses of SBPs following protein separation by SDS-PAGE (SI Fig. S1).

### Purification of complex I from *P. denitrificans*

**Figure 1:**
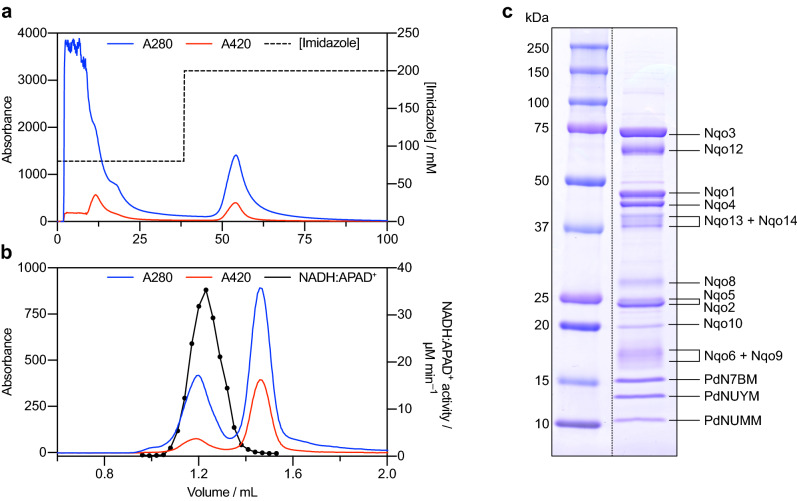
Purification and subunit composition of *P. denitrificans* complex I. (**a**) Typical Ni-affinity chromatography trace produced using a HisTrap HP column. The column was washed with 80 mM imidazole and protein eluted with 200 mM imidazole. (**b**) Typical size exclusion chromatography trace using a Superdex 200 increase 5/150 GL column. Complex I was identified by measuring the relative NADH:APAD^+^ activity in eluted fractions (black trace). (**c**) SDS-PAGE analysis of purified *P. denitrificans* complex I. Two full-length lanes are shown from a single gel; the image has been cut and they have been moved to be adjacent to each other. The original image is shown in SI Fig. S6. Individual complex I subunits were identified and assigned by excising each band, treating the sample with trypsin and analyzing the resultant peptides by mass spectrometry. Peptides were assigned to a subunit/protein by peptide mass fingerprinting.

To purify *P. denitrificans* complex I, membranes were prepared from *P. denitrificans* cells grown aerobically on LB medium. The conditions for membrane solubilization with *n*-dodecyl β-D-maltoside (DDM) and the buffer compositions were based on those of Yip et al., with Bis–Tris replaced by MES and our affinity chromatography buffers supplemented with 0.02% asolectin^26,42^. Following solubilization, the proteins were loaded onto a Ni-affinity column and complex I eluted using 200 mM imidazole (see "Materials and methods" for details) (Fig. 1a). Complex IV coeluted with complex I, so the eluent was further separated by size exclusion chromatography; only the first peak exhibited complex I flavin site activity (NADH:APAD^+^ oxidoreduction) and it was thus assigned to complex I (Fig. 1b). The complex I peak eluted at the volume expected for intact *Pd*-CI, as predicted from the eluted volumes of standard proteins given in the manufacturer's guide (Cytiva). The purified enzyme contained 1.50 ± 0.13 Q_10_ molecules per complex I (S.E.M., n = 3 samples from three independent cultures and purifications), which is similar to the ubiquinone content measured for the purified *E. coli* enzyme (1.3 ± 0.1)^43^. The *Pd*-CI was analyzed by SDS-PAGE and prominent bands were excised, digested with trypsin and the proteins identified by mass spectrometry analyses (Fig. 1c). All the core complex I subunits were identified, apart from Nqo7 and Nqo11, which are likely missed from the analysis due to their small and hydrophobic nature (SI Table S1). Further to the core subunits, the three previously identified supernumerary subunits (PdNUMM, PdNUYM, PdN7BM) were also detected^26^. Our mass spectrometry analyses also identified several additional proteins with varying degrees of confidence (SI Table S1). Most of these are likely minor contaminants and unlikely to be associated with complex I, such as elongation factor Tu, transcription termination factor Rho and subunits from other respiratory complexes. A small number of other proteins were identified, notably a protein-L-isoaspartate *O*-methyltransferase (23.5 kDa), which cannot be either excluded from or assigned to complex I at the current time. Structural studies will be required to determine whether these are impurities or unique subunits associated with the *P. denitrificans* enzyme.

### EPR characterization of purified complex I

**Figure 2:**
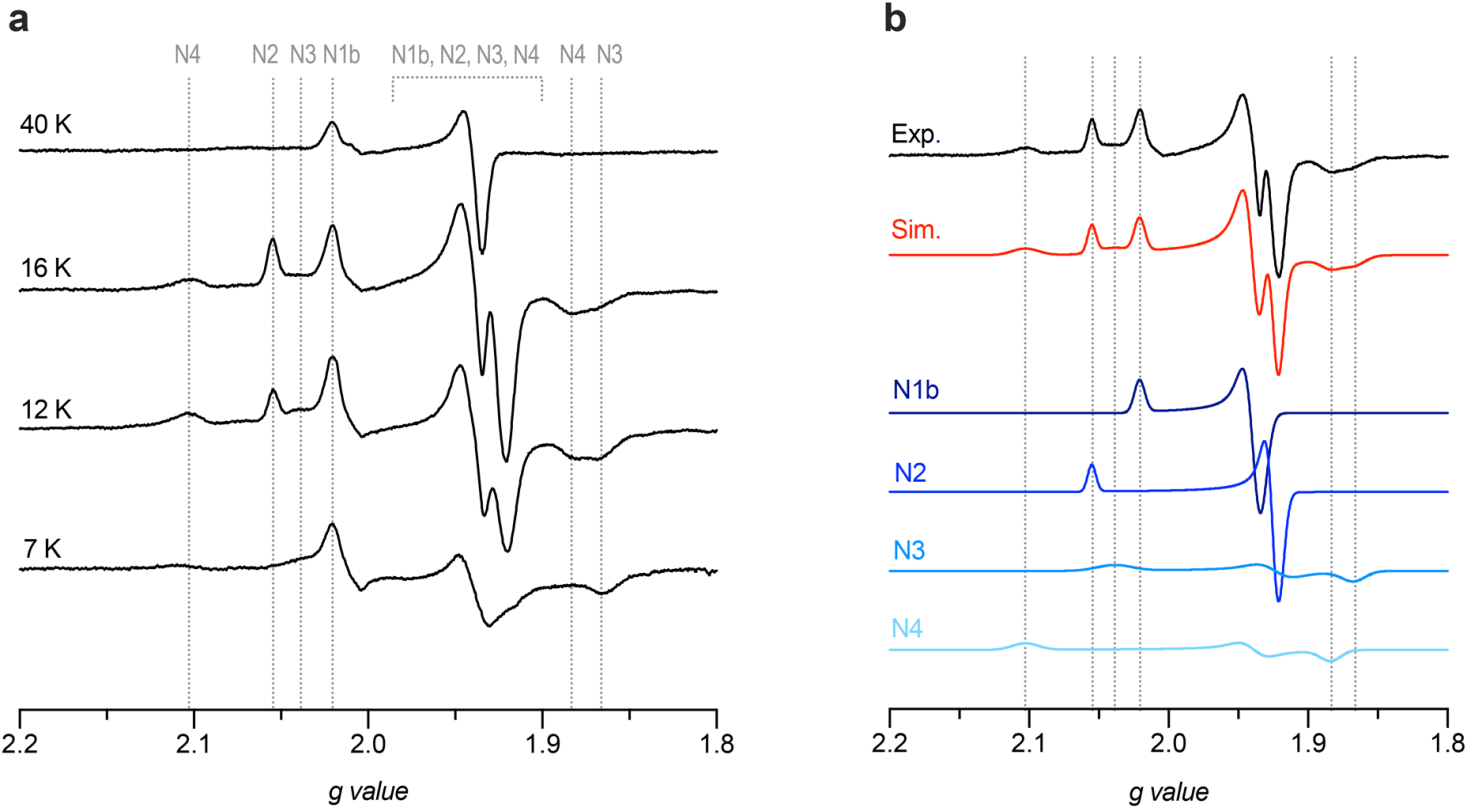
EPR spectra of NADH-reduced purified complex I from *P. denitrificans*. (**a**) Complex I (5.8 mg mL^–1^) was reduced anaerobically with 15 mM NADH. EPR spectra were recorded at 100 kHz modulation frequency with a modulation amplitude of 7 G and a microwave power of 2.02 mW at the temperatures indicated. Vertical lines correspond to the *g* factors for the individual FeS clusters. (**b**) Simulation of the EPR spectrum of purified *Pd*-CI at 16 K. The total simulation of the combined FeS cluster signals is shown in red, with the individual simulations labelled according to the established nomenclature. FeS clusters N1b-N4 were simulated at a 1:1 ratio. Simulation parameters are given in SI Table S2.

In order to compare the electron transfer pathways in *Pd-*CI and its mammalian counterpart, electron paramagnetic resonance (EPR) spectroscopy was performed. Previous EPR studies of *Pd*-CI have been limited to membrane particles^44–46^, which make deconvolution of the CI specific signals difficult. EPR spectra of the NADH-reduced purified *Pd*-CI were measured at different temperatures (Fig. 2a). The spectra clearly show the presence of four reduced FeS clusters (N1b, N2, N3 and N4), confirming the integrity of the hydrophilic domain in the purified enzyme. At 40 K, a single axial FeS cluster corresponding to the slow-relaxing [2Fe-2S] N1b is observed. As the temperature is decreased, additional [4Fe-4S] FeS signals appear in accordance with the established relaxation properties for mammalian complex I (N1b < N2 < N3 < N4)^47,48^. At 7 K, the FeS signals from N2 and N4 are largely saturated. The fast-relaxing N5 cluster was not observed during these experiments as high power and low temperature are typically required to resolve its signal; however, overexpression of the *P. denitrificans* Nqo3 (NDUFS1) subunit in *E. coli* has previously given rise to a signal attributable to N5^49^.

A simulation of the *Pd*-CI EPR spectrum recorded at 16 K, using an equal stoichiometry of the four observed FeS clusters, is shown (Fig. 2b). This ‘signature’ spectrum for *Pd-*CI shows remarkable similarity to that observed for mitochondrial complex I from *Y. lipolytica*, which could also be simulated with an equal stoichiometry for the same four reduced clusters^50^. In contrast, mammalian complex I exhibits sub-stoichiometric reduction of N1b when NADH is used as a reductant, owing to its low reduction potential^48^. As in both of these mitochondrial complexes, but unlike in complex I from *E. coli*, the [2Fe-2S] cluster in Nqo2 (NDUFV2), referred to as N1a, is not reduced in NADH treated *Pd*-CI. In *E. coli* complex I, N1a is a high potential cluster that is reduced during turnover whereas N3, which is fully reduced by NADH in both mitochondrial and *Pd*-CI, appears to be reduced sub-stoichiometrically^51,52^. The EPR signal for N1a has thus far only been observed in overexpressed *Pd*-Nqo2 reduced with sodium dithionite, with rhombic geometry and characteristic *g* values of 2.00, 1.94 and 1.92^53,54^ that are clearly not present in Fig. 2a. The absence of the reduced N1a signal is consistent with its reduction potential being below that of NADH^54,55^, and with data on mammalian complex I that show reduction of N1a only in the dithionite-reduced flavoprotein subcomplex, not in the intact enzyme^48,55,56^. The properties of the [2Fe-2S] N1a cluster and pattern of FeS cluster reduction thus further establish the close similarity between *Pd*-CI and mitochondrial complex I.

### Catalysis by isolated (detergent-solubilized) *P. denitrificans* complex I

**Figure 3:**
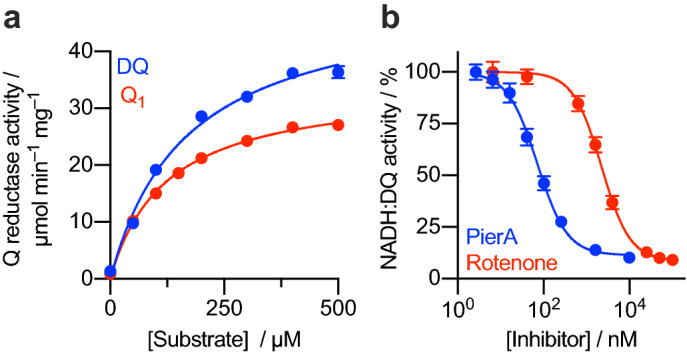
Characterization of purified complex I from *P. denitrificans.* (**a**) *K*_M_ curve for DQ and Q_1_ substrates for a typical sample of soluble complex I. The *K*_M_ values for DQ and Q_1_ are 169 ± 15 and 124 ± 8 µM, respectively (± S.E. of the fit). (**b**) Piericidin A and rotenone IC_50_ titration curves for soluble complex I, using DQ as the substrate. The IC_50_ values of piericidin A and rotenone are 72 ± 7 nM and 2226 ± 185 nM, respectively (± S.E. of the fit).

The integrity and catalytic competence of the complex I isolated from *P. denitrificans* were determined by measuring its catalytic activity. The optimized assay conditions previously described for *Pd*-CI by Yip et al. were confirmed here^26^: the activity was highest in buffer at pH 6.5 and in the presence of divalent cations, Mg^2+^ or Ca^2+^ (SI Fig. S2). The addition of sucrose (to make the buffer more hypertonic) had no effect on activity. Under optimal assay conditions, our preparations of *Pd*-CI were able to catalyze the reduction of 200 µM decylubiquinone (DQ) by NADH at typically 21.9 ± 4.2 µmol min^–1^ mg^–1^ (~ 204 s^–1^, S.D. from 11 independent purifications). For comparison, the purified bovine, ovine and *Y. lipolytica* enzymes have been reported to catalyze the reduction of 200 µM DQ at 22.2–24.7 µmol min^–1^ mg^–1^ (~ 390 s^–1^)^57^, 5–6 µmol min^–1^ mg^–1^ (~ 90 s^–1^)^20^ and 13.9 µmol min^–1^ mg^–1^ (~ 208 s^–1^)^17^, respectively, and the bacterial enzyme from *E. coli* to catalyze reduction of 100 µM DQ at 20–25 µmol min^–1^ mg^–1^ (~ 206 s^–1^)^43,58^. The activities of two soluble quinone analogues, Q_1_ and DQ, were then compared on the same enzyme preparation (Fig. 3a). *Pd*-CI catalyzed DQ reduction to higher rates than Q1 reduction, however, the high *K*_M_ values observed for both DQ (169 ± 15 µM) and Q_1_ (124 ± 8 µM) suggest that neither is a good substrate for the *P. denitrificans* enzyme. It is worth noting that DQ and Q_1_ have limited solubility in aqueous solution and so activity data recorded at high substrate concentrations, where they tend to aggregate or form micelles, may not provide an accurate picture. Unless otherwise stated, activities reported here were measured using 200 µM DQ, to match standard data on the bovine enzyme^57^.

To estimate the Q-reductase activity of native *Pd*-CI in the membrane, and thus the highest possible activity we may achieve for the purified enzyme, we compared activities in the membranes with those of the soluble form. The membranes and isolated enzyme were able to catalyze flavin-catalyzed NADH:APAD^+^ oxidoreduction at 0.497 ± 0.015 µmol min^–1^ mg^–1^ and 9.98 ± 0.05 µmol min^–1^ mg^–1^, respectively (S.E.M. of three technical replicates). By assuming that the flavin site activity is fully retained during purification, and that the only NADH:APAD^+^ activity in the membrane is from complex I, the complex I content of the membranes was estimated at 5.0 ± 0.2% (by protein mass). The membranes were able to catalyze NADH:O_2_ oxidoreduction at 2.02 ± 0.09 µmol min^–1^ mg^–1^, which translates to a Q-reductase activity of 40 ± 2 µmol min^–1^ mg^–1^, based on the estimated complex I content. Therefore, our rate of DQ reduction suggests we have retained 54 ± 11% of the Q-reductase activity in our preparation – although greater activities may be observed at higher concentrations of DQ, or by using the native Q_10_ as the electron acceptor. Overall, these data provide clear evidence that our preparation of *Pd*-CI is highly active for NADH:ubiquinone oxidoreduction.

The sensitivity of the purified enzyme to the canonical complex I inhibitors piericidin A and rotenone was then determined (Fig. 3b). The NADH:DQ activity was inhibited ≥ 90% by piericidin A and rotenone. However, the enzyme was more sensitive to piericidin A (IC_50_: 72 ± 7 nM) than rotenone (IC_50_: 2226 ± 185 nM). A previous study has also shown that, unlike the mitochondrial enzyme, *Pd*-CI is poorly inhibited by rotenone^59^. Surprisingly, *Pd*-CI purified by Yip et al. was reported to exhibit similarly high sensitivities to both rotenone (IC_50_: 170 nM) and piericidin A (IC_50_: 100 nM)^26^. These values do not agree with our data or with previous data, despite our similar buffer and assay conditions. The enzyme preparation of Yip et al. was reported to have a lower activity (15.0 µmol min^–1^ mg^–1^) than ours (21.9 µmol min^–1^ mg^–1^), although it was assayed with a lower concentration of DQ. In addition, we note our preparation time was much shorter (hours *vs*. days), so it is possible we have maintained greater structural integrity at known rotenone binding sites in the Q-channel and near the transverse helix^20^.

Finally, we assessed the rate of H_2_O_2_ generation by our purified enzyme using the horseradish peroxidase-dependent oxidation of Amplex Red to resorufin (see "Materials and methods"). The Amplex Red system detects the H_2_O_2_ formed, either directly or by the dismutation of superoxide, assisted by the presence of SOD. H_2_O_2_ was generated at 138.6 ± 1.1 nmol min^–1^ mg^–1^ (78 min^–1^) using 30 µM NADH to activate superoxide/H_2_O_2_ production at the flavin site, approximately four times faster than generated by the purified complex I from bovine heart mitochondria (21.1 ± 2.9 nmol min^–1^ mg^–1^, 21 min^–1^)^60^. However, the different pH values at which the two assays were performed (pH 6.5 *vs*. pH 7.5) may also influence the rate of H_2_O_2_ production^60^.

### Optimization of the reconstitution of *Pd*-CI into liposomes

To study proton pumping of wild-type and mutated *Pd*-CI in a controlled minimal membrane environment, the purified enzyme needs to be reconstituted into a compartmentalized membrane system that supports formation of a ∆p. We have established protocols for the assembly of proteoliposome (PL) systems comprising mitochondrial complex I, ubiquinone Q_10_, and *Trypanosoma brucei brucei* alternative oxidase (AOX), which re-oxidizes ubiquinol Q_10_H_2_ to ubiquinone Q_10_ and reduces oxygen to water^61,62^. The system was later expanded to couple ∆p formation by complex I to ATP synthesis by further incorporating *E. coli* ATP synthase into the proteoliposomes^63^. AOX is a monotopic membrane protein that associates spontaneously to the liposomal membrane and thus it was added after reconstitution (to the mature proteoliposomes) in all experiments. Here, we adapted a protocol optimized for mammalian CI^63^ for the efficient incorporation of *Pd*-CI. As soluble *Pd*-CI was found to be most active at pH 6.5 (SI Fig. S2a), the pH of the reconstitution buffer was adjusted to pH 6.5 with MES replacing MOPS as the buffering component. Reconstitution of *Pd*-CI into our standard liposomes that mimic the mitochondrial inner membrane lipid composition (80:10:10 dioleoyl phosphocholine (DOPC):dioleoyl phosphoethanolamine (DOPE):tetraoleoyl cardiolipin (TOCL), in % (w/w)) for the reconstitution of mitochondrial CI^61,63,64^, resulted in only low activities of 7.08 ± 0.63 μmol NADH min^-1^ mg^-1^ (n = 8) compared to NADH:Q_10_ oxidoreduction activities of up to 40 ± 2 µmol min^–1^ mg^–1^ estimated for *Pd*-CI in membranes. Thus, we amended the lipid mixture to more closely mimic the reported lipid composition of exponentially growing *P. denitrificans*^65^, to 52 dioleoyl phosphoglycerol (DOPG):37 DOPC:8 DOPE:3 TOCL. Although this did not affect the activity of reconstituted *Pd*-CI (SI Fig. S3a) we adopted it for all further experiments. Significantly increased activities were subsequently observed (SI Fig. S3a) when 250 mM sucrose was included in the reconstitution and catalytic assay buffers (see below). Next, the amount of AOX was varied to achieve optimal turnover (SI Fig. S3b). The optimal amount of AOX was found to be 40–80 µg mL^–1^ AOX per 0.5 µg mL^–1^ outward facing *Pd*-CI, which translates to 1200–2400 molecules of AOX added (to the cuvette) per *Pd*-CI. These optimized conditions were used for all further experiments.

### Characterization of *Pd*-CI proteoliposomes

The reconstitution protocol optimized for *Pd*-CI activity led to a random orientation of *Pd*-CI in the membrane, with 46.7 ± 16.8% (n = 9, S.D., nine reconstitutions using *Pd*-CI from four different preparations) of the NADH:APAD^+^ oxidoreductase activity on the outside of the proteoliposomes, reflecting around half of the *Pd*-CI being inserted in an outward facing orientation. As NADH is membrane impermeable, the inward facing CI is catalytically silent in kinetic assays. The average protein retention (relative to the amount added at the start of the preparation) was determined as 36.9 ± 12.7% (n = 10, S.D.). The average NADH:O_2_ activity (for outward facing *Pd*-CI) for a standard preparation was 24.5 µmol min^–1^ mg^–1^ ± 8.4 µmol min^-1^ mg^-1^ (n = 11, S.D.). However, the NADH:O_2_ activity varied substantially, from, 13.8 to 41.5 µmol min^–1^ mg^–1^, with more than 50% of the preparations giving activities between 22.6 and 26.6 µmol min^–1^ mg^–1^. Only small respiratory control ratios (RCRs) of 1.2 ± 0.2 (n = 8, S.D.), similar to our observations for bovine CI PLs^61^, were measured. Comparing the average NADH:O_2_ activity to our estimate for the Q-reductase activity of native *Pd*-CI in the membranes, 61 ± 15% of the total activity is retained during purification and reconstitution of *Pd*-CI.

**Figure 4:**
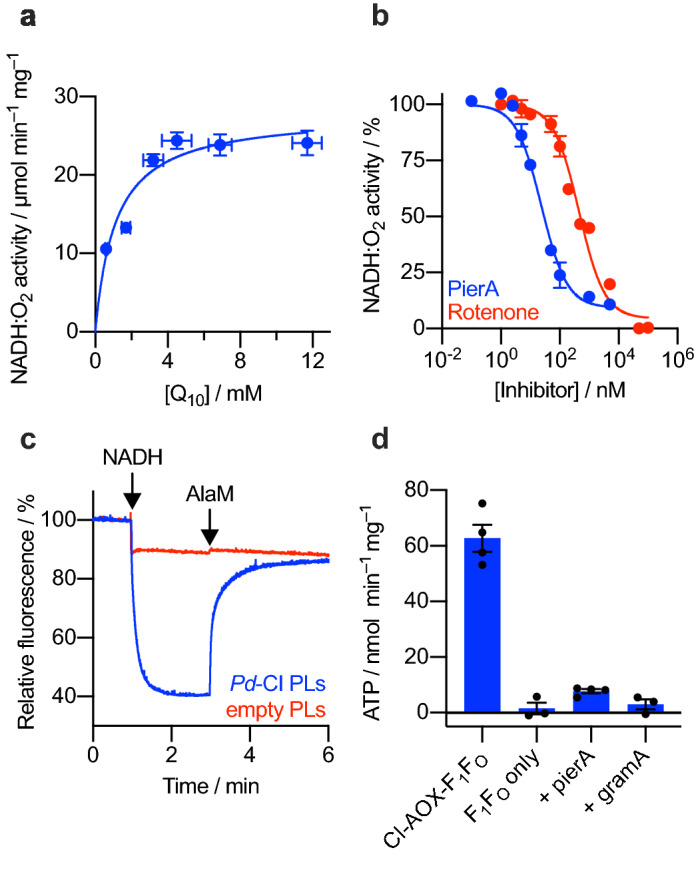
Characterization of *Pd*-CI in liposomes. (**a**) *K*_M_ curve for Q_10_ in proteoliposomes. The *K*_M_ and *V*_max_ values were determined to be 1.1 ± 0.2 mM and 27.9 ± 1.2 µmol min^–1^ (mg CI)^–1^, respectively (± S.E.M. of the fit). (**b**) Inhibition by piericidin A and rotenone. The measured IC_50_ values for piericidin A and rotenone were 24 ± 3 nM and 452 ± 48 nM, respectively (± S.E. of the fit). (**c**) Proton pumping measured by an ACMA fluorescence quench assay. Proton pumping was initiated by addition of 1 mM NADH and proteoliposomes uncoupled by addition of 25 µg mL^–1^ alamethicin (AlaM). Liposomes without *Pd*-CI were added as a control. (**d**) Coupling *Pd*-CI catalysis to ATP synthesis. *Pd*-CI was co-reconstituted in liposomes with *E. coli* ATP synthase (CI-AOX-F_1_F_O_) and NADH-coupled ATP production was measured as luminescence using a luciferase-based assay. No ATP was generated by proteoliposomes containing only ATP synthase (F_1_F_O_ only) while addition of 2 μM piericidin A (pierA) or 20 μg mL^–1^ gramicidin A (gramA) inhibited ATP synthesis. Alternative oxidase (AOX) was directly added to the assay mixture at 20 µg mL^–1^ in panels (**a**–**c**) and at 5 µg mL^–1^ in panel (**d**). See “Materials and methods” for the assembly of proteoliposomes and other assay conditions.

The *K*_M_ for the reduction of native ubiquinone Q_10_ was measured in *Pd*-CI PLs (Fig. 4a, in which all the data points are from individual reconstitutions and determinations). The *K*_M_ value of 1.1 ± 0.2 mM for ubiquinone Q_10_ is within the range (0.48–3.94 mM)^61–63^ of experimentally determined Q_10_ *K*_M_ values for bovine CI, indicating similar affinities for Q_10_ for both enzymes. The inhibitor titrations for piericidin A and rotenone carried out with the soluble enzyme were repeated in *Pd*-CI proteoliposomes to investigate how the membrane affects the inhibition (Fig. 4b). The IC_50_ values for both inhibitors were decreased in PLs from 2226 ± 185 nM to 452 ± 48 nM and from 72 ± 7 nM to 24 ± 3 nM for rotenone and piericidin A, respectively. This is likely due to the smaller hydrophobic phase volume in the PL measurements, which concentrates the inhibitor in the vicinity of the enzyme. However, the same trend of weaker inhibition by rotenone than piericidin A was confirmed. *Pd*-CI appears to not bind canonical inhibitors of complex I, particularly rotenone, as strongly as the mitochondrial enzymes do. Understanding the fundamental differences in inhibitor binding will require structural work and comparison to known piericidin A and rotenone-bound complex I structures^18–20^.

Next, it was tested whether NADH oxidation is coupled to proton pumping in liposome-reconstituted *Pd*-CI by using the standard fluorescence quench assay, ACMA (9-amino-6-chloro-2-methoxyacridine) quenching (Fig. 4c)^61^. Addition of NADH to *Pd*-CI-AOX PLs led to a rapid quench of the fluorescence signal indicating formation of a ΔpH. Addition of the uncoupler alamethicin dissipated the ΔpH, while empty liposomes did not show any fluorescence quench upon NADH addition. The addition of piericidin A to the liposomes also prevented fluorescence quenching after NADH addition by inhibiting catalysis and proton pumping (SI Fig. S4). In a separate experiment, *Pd*-CI was co-reconstituted with *E. coli* ATP synthase into liposomes to test if a substantial ∆p is being produced by the *Pd*-CI (Fig. 4d). Indeed, addition of NADH to CI-AOX-F_1_F_O_ PLs coupled NADH oxidation (and ubiquinone reduction) to proton pumping, establishing a ∆p high enough to drive ATP synthesis. No ATP was produced when NADH was added to proteoliposomes containing only *E. coli* ATP synthase, and ATP synthesis in CI-AOX-F_1_F_O_ PLs was inhibited by piericidin A, or when the ∆p was collapsed by the uncoupler gramicidin A. Together these results indicate specific reconstitution of intact *Pd*-CI into sealed liposomes, which couple NADH oxidation to ∆p formation, and demonstrate its proton pumping capability. Therefore, our reconstitution protocol can be applied to aid study of the proton pumping mechanism of mutant and wild-type CI in future experiments.

### A genetic system to study complex I variants

**Figure 5:**
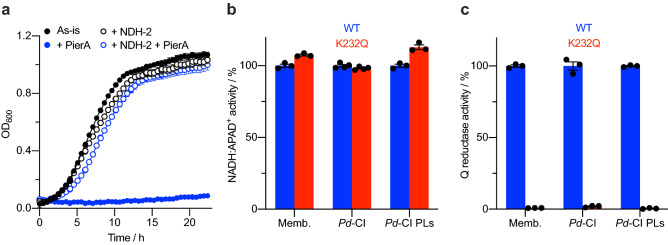
Creation of a catalytically inactive complex I variant. (**a**) Growth of wild-type *P. denitrificans* in succinate minimal media^40,41^. Cells were grown with/without 10 mM taurine to induce NDH-2 expression and with/without 5 µM piericidin A to inhibit complex I and cell growth. (**b**) Comparison of the complex I flavin site activity (NADH:APAD^+^) of wild-type and the K232Q^Nqo13^ variant measured in three different contexts. Membrane activities were measured using dNADH due to the expression of NDH-2 during cell growth. The specific activities have been normalized to the wild type activities; in membranes (0.527 ± 0.006 µmol min^–1^ mg^–1^, S.E.M. n = 3), purified CI (12.13 ± 0.14 µmol min^–1^ mg^–1^, S.E.M. n = 4), and proteoliposomes (10.71 ± 0.12 µmol min^–1^ mg^–1^, S.E.M. n = 3). (**c**) Comparison of the quinone reductase activity (Q_10_ or DQ) of wild-type and the K232Q^Nqo13^ variant measured in three different contexts. The specific activities have been normalized to the wild type activities (minus the average piericidin A insensitive rates) in membranes (2.41 ± 0.02 µmol min^–1^ mg^–1^, S.E.M. n = 3), purified enzyme (19.73 ± 0.058 µmol min^–1^ mg^–1^, S.E.M. n = 3), and proteoliposomes (41.45 ± 0.17 µmol min^–1^ mg^–1^, S.E.M. n = 3).

Complex I is essential for the survival of *P. denitrificans* cells, preventing the creation of severely deleterious complex I variants. To overcome this issue, Finel and coworkers inserted the alternative NADH dehydrogenase (NDH-2) from *E. coli* directly into the *P. denitrificans* genome to compensate for loss of activity in variants generated in the Nqo8 (ND1) subunit^66,67^. Here, we introduced *ndh2* from *E. coli* into *P. denitrificans* on the inducible expression vector pLMB509^68^. Using this approach, we could control whether or not NDH-2 was expressed by including the inducer, taurine, in the growth medium, allowing us to generate deleterious complex I mutants. The tunability of the system means that we are also able to turn off NDH-2 expression to prevent it interfering with activity measurements on non-deleterious mutants. The complex I specific substrate deaminoNADH (dNADH), which is not oxidized by NDH-2, can be used in those cases where NDH-2 is required for cell growth. To show that expression of NDH-2 allows cells to grow when complex I catalysis is severely compromised, we grew *P. denitrificans* cells in succinate media and inhibited complex I with piericidin A in both cells expressing or not expressing NDH-2 (Fig. 5a). While the addition of piericidin A severely limited cell growth, the expression of NDH-2 rescued it to near untreated levels: the cells remain viable despite a severely compromised complex I.

To demonstrate the application of our system, we generated a complex I variant that we expected would abolish catalytic activity. We chose to remove the charge from the conserved charged residue Lys232 in the Nqo13 (ND4) subunit by mutation to a Gln residue (K232Q^Nqo13^). Mutation of the equivalent residue in *E. coli* to Ala rendered complex I inactive^69^. The mutation was generated using a method similar to that used to insert the affinity purification tag. Crucially, NDH-2 was expressed throughout the growth protocol, essential for successful mutagenesis. The K232Q^Nqo13^ mutant was confirmed by sequencing and the cells were only able to grow when expressing NDH-2. Membranes were prepared for the K232Q^Nqo13^ mutant from cells expressing NDH-2 and the activities of the membranes were measured using dNADH. While the dNADH:APAD^+^ activity was similar between the wild-type (i.e. *Pd*-Nqo5^His6^) and K232Q^Nqo13^, the quinone reductase activity was abolished in the mutant (Fig. 5b,c). We further characterized the mutant by first purifying K232Q^Nqo13^ and then reconstituting it into liposomes. The K232Q^Nqo13^ mutant purified as normal, showed essentially identical elution profiles to wild-type on the size exclusion column, and a matching band pattern to wild-type in SDS-PAGE analyses (SI Fig. S5). These observations suggest that the structural integrity of the mutant was maintained throughout purification and its loss of activity has a mechanistic basis rather than being due to structural instability. Both the soluble enzyme and the proteoliposomes showed similar relative activities to those measured in membranes (Fig. 5b,c) and consequently proteoliposomes of the K232Q^Nqo13^ mutant showed no proton pumping activity as measured by ACMA (SI Fig. S4).

## Discussion

In order to answer long-standing questions about the energy coupling and proton translocation mechanisms of respiratory complex I, functional and structural analyses should be combined with targeted mutagenic interrogation of specific residues and protein features. The α-proteobacterium *P. denitrificans*, a close relative of the protomitochondrion, is thus a prime candidate for a model system that could be exploited to unlock the catalytic mechanism of mitochondrial complex I, as it enables all three approaches. In addition to the similarities *Pd*-CI possesses with the mitochondrial enzyme that are uncommon in other bacterial models (Q_10_ as an electron acceptor; higher subunit sequence homology; supernumerary subunits and complex I-containing supercomplexes), it is possible to form well-coupled SBPs as a powerful tool for insightful biophysical and biochemical characterization. In particular, using SBPs, we have already established a method that can be used to determine the number of protons translocated across the membrane by complex I for every NADH oxidized (the proton stoichiometry)^35,36^. Thus *P. denitrificans* provides an exciting platform to combine site-directed mutagenesis with biochemical and biophysical measurements essential for gaining mechanistic insight into complex I.

Here, we establish the potential of *P. denitrificans* as a suitable bacterial model for mitochondrial complex I. First, an affinity purification tag was engineered onto the Nqo5 subunit, allowing reproducible and straightforward purification of intact, catalytically active complex I. The reconstitution of the detergent-solubilized complex into liposomes was then optimized, demonstrating that the complex I retained proton pumping activity. The two protocols allow *Pd*-CI to be studied in both its soluble form and when reconstituted in a controllable minimal membrane system, providing a complementary perspective to that which can be gained from well-established measurements in SBPs. Second, we created a strain of *P. denitrificans* that is fully genetically tractable to complex I mutagenesis. As complex I is essential to growth of *P. denitrificans*, its mutagenesis was otherwise limited by the inability to create catalytically inactive or compromised variants that would kill the cell. By introducing NDH-2 from *E. coli* into *P. denitrificans* on a taurine-inducible expression plasmid, we were able to show cell survival and growth when complex I was compromised, by expressing NDH-2. By creating a catalytically inactive complex I variant by a mutation in the Nqo13 subunit we demonstrate the power of our approach, both in being able to generate mutations in the membrane domain, not currently routinely possible in mitochondrial models, and being able to target even essential residues, regardless of their loss of activity.

Despite the many advantages of using *P. denitrificans* as a model for mitochondrial complex I, some current technical limitations should be noted. Our approach generates unmarked mutations in the *P. denitrificans* genome which, while beneficial as it ensures complex I variants will be constitutively expressed, is more technically challenging/slower than introducing mutations on a gene expressed on a plasmid, as employed in *Y. lipolytica*^39^ or *E. coli*^21,30^. Further to this, the high GC content of the *P. denitrificans* genome requires careful consideration during even basic cloning procedures. Perhaps the most obvious limitation is that there is no *P. denitrificans* complex I structure yet available, a crucial element that is required to fully realize its potential as a model for combining both functional and structural data with mutagenesis. Although we have demonstrated we can isolate intact and highly active complex I from *P. denitrificans*, the lack of stabilizing supernumerary subunits in the bacterial enzyme likely decreases its stability compared to its mitochondrial counterparts. This possibility may explain the relative lack of bacterial complex I structures solved by cryo-EM to date. It is known that *P. denitrificans* complex I dissociates under Blue-Native PAGE conditions^27^ despite remaining intact during solubilization and in our two-step purification, and the freezing methods used in cryo-EM grid preparation may also be detrimental. A potential alternative route to the *P. denitrificans* complex I structure may be isolation of the respiratory supercomplex, which is known to stabilize *Pd*-CI and can be visualized on Blue-Native PAGE^27^. Although we are confident in expecting the enzyme to have a similar overall architecture to current models, and the positions of key conserved residues to be in similar configurations, pursuit of the *P. denitrificans* complex I structure will be both beneficial and complementary to the work described here.

As described above, complex I from *P. denitrificans* possesses many similarities to the mitochondrial enzyme, making it an attractive model system. However, there are also fundamental differences between the two that can be considered both beneficial and disadvantageous. An important property of mitochondrial complex I is that it can undergo an active/deactive (A/D) transition^13,57,70^ in which the deactive state cannot catalyze quinone reduction and the enzyme is considered to be in an off-pathway resting state. The deactivate state is identified biochemically by the binding of *N*-ethylmaleimide (NEM) to a conserved cysteine residue in Nqo7 (ND3) that is only exposed in the deactive state, locking it in this state. *P. denitrificans* has not been observed to undergo an A/D transition^46,70^ and here we also tested whether *Pd*-CI could be deactivated in membranes by assaying the membrane activity in control and heat-treated membranes (conditions which deactivate the mouse and bovine enzymes^13,57^) with and without NEM. We observed no differences in activity to suggest the enzyme is able to form an (NEM-sensitive) deactive state (SI Table S3). Hence, *Pd*-CI cannot be used as a model to assess or investigate the A/D transition that occurs in mitochondria, which may be a unique feature of the mitochondrial enzyme. However, *Pd*-CI can instead be considered a model for the ‘active’ mitochondrial enzyme, which is an advantage for studies of the mechanism of catalysis as the effects of mutations in catalytically relevant residues can be deconvoluted from possible effects on the A/D transition in the mitochondrial enzyme. In this regard, as well as in many others, *P. denitrificans* is a powerful system for probing the mechanism of complex I catalysis.

## Materials and methods

### Chemicals

All chemicals were purchased from Sigma-Aldrich unless stated otherwise.

### Creation of bacterial strains

#### Insertion of NDH-2 into *P. denitrificans*

The NDH-2-encoding gene from *E. coli* (*ndh2*) was optimized for the codon usage in *P. denitrificans* and the construct synthesized by GENEWIZ®. Two restriction sites were added to the 5ʹ (*NsiI*) and 3ʹ (*SacI*) ends of the construct, which was then digested and inserted into the expression vector pLMB509 kindly provided by Dr Andrew Gates (University of East Anglia)^68^. The expression vector used here was a modified version that has had the GFP marker removed. The His_6_-tag present in the vector was removed during the *ndh2* insertion. The vector was transformed into the MFD*pir E. coli* donor strain and subsequently conjugated into the *Pd*1222-∆Hy parental strain^35^ of *P. denitrificans* at a 10:1 donor to recipient ratio. After two days of incubation at 30 °C on LB plates containing 0.5 mM diaminopimelic acid, successful conjugation was selected for by plating serial dilutions onto LB agar containing rifampicin (50 µg mL^–1^) and gentamicin (20 µg mL^–1^).

#### Generation of the affinity-tagged complex I strain

The location and design for the affinity tag replicated the purification tag previously engineered onto *Y. lipolytica* complex I^38,39^. The affinity tag was designed to contain six histidine residues attached to the C-terminus of the Nqo5 subunit by six alanine linker residues. The insertion of the affinity tag onto the C-terminus of the Nqo5 subunit of *P. denitrificans* complex I was achieved using a similar strategy as described previously for creating deletions in the *P. denitrificans* genome^35,40,41^. Insertion cassettes were designed containing two sequences homologous to regions on either side of *nqo5* (Pden 2248, nt 2,250,063–689). The first homologous flanking region of 1292 bp began at nucleotide position 2,248,772 and extended to position 2,250,065 to include the STOP codon of the *nqo5* gene. The sequence for the His_6_Ala_6_-tag (5ʹ-GTG ATG GTG ATG ATG ATG CGC GGC TGC CGC GGC GGC-3ʹ) was then inserted to become the new C-terminal sequence of *nqo5*. The next flanking region contained the entire *nqo5* gene (minus the STOP codon) and extended for a further 1071 bp to nucleotide position 2,251,760. This second flanking region was followed by a kanamycin (*kan*^*R*^) selection marker of 815 bp length. *Eco*RI restriction sites were added to the end of the construct and any *Eco*RI sites within the construct itself were removed by silent mutagenesis. The construct was assembled by GENEWIZ® and then inserted into the *lacZ*-containing pRVS1 suicide plasmid via an *Eco*RI restriction site. The plasmid was transformed into the MFD*pir E. coli* donor strain and conjugated into the *Pd*1222-∆Hy parental strain containing the *ndh2* gene on pLMB509. NDH-2 was expressed throughout conjugation and homologous recombination by including taurine (10 mM) in all media. Successful conjugation and first recombination events were selected by using LB-agar plates containing 50 µg mL^–1^ kanamycin. Successful colonies were then plated on X-gal (200 µg mL^–1^), and white colonies were selected as positive for the second recombination event, and screened by PCR amplification of the DNA sequence across the *nqo5* gene to confirm the expected insertion of base pairs. The integrity of the strain was determined by full sequencing of all complex I subunits. Here, we refer to the His_6_-tag strain as *Pd*-Nqo5^His6^, or as the ‘wild-type’ strain in comparisons with the K232Q^Nqo13^ variant.

#### Generation of the K232Q^Nqo13^ complex I variant

The K232Q^Nqo13^ complex I variant was created in our *Pd*-Nqo5^His6^ strain that also contained the *ndh2* gene on pLMB509. The same procedure as above was followed to generate the mutation, ensuring NDH-2 was expressed continuously throughout the protocol by the inclusion of 10 mM taurine in all media. The point mutation cassettes were designed to include homologous flanking regions 1000 bp upstream and downstream of *nqo13* (Pden_2232). The kanamycin (*kan*^*R*^) selection marker followed the second flanking region. The lysine residue in Nqo13 at amino acid position 232 was mutated to a glutamine (AAG to CAG). The creation of the mutant was confirmed by sequencing.

### Bacterial growth and membrane preparations

#### Succinate media growth curves

Colonies from the *Pd*-Nqo5^His6^ strain were picked and grown for 24 h in 40 mL succinate minimal media with/without 10 mM taurine (30 °C, 225 rpm); the culture was then used to inoculate 200 µL aliquots of succinate minimal medium in a 96-well plate to a starting optical density of 0.02 (at 600 nm). Media were supplemented with/without 10 mM taurine and with/without 5 µM piericidin A and each experiment was carried out in quadruplicate. Plates were incubated at 30 °C with 200 rpm orbital shaking in a CLARIOstar *Plus* microplate reader (BMG Labtech) and the optical density was recorded every 30 min. The minimal medium used was adjusted to pH 7.2 and contained 50 mM succinate, 9.35 mM NH_4_Cl, 2 mM MgSO_4_, 0.07 mM CaCl_2_, 0.29 mM KH_2_PO_4_, 0.69 mM K_2_HPO_4_, 25.2 mM Na-Hepes, 19.6 μM Na_2_-EDTA, 9 μM FeSO_4_, 0.1 μM MnCl_2_, 0.8 μM CuCl_2_, 1 μM Na_2_MoO_4_ and 2.5 μM ZnCl_2_^40,41^.

#### Membrane preparations

Colonies from the *Pd*-Nqo5^His6^ strain were picked and grown for 24 h in 50 mL LB (30 °C, 225 rpm) then 12 × 500 mL flasks of LB medium were each inoculated with 500 µL of preculture. Flasks were grown for 16–20 h (30 °C, 225 rpm) and harvested at late log phase when the OD_600_ was 3.5–4.5. Cells were collected by centrifugation in a Sorvall RC 12BP centrifuge and resuspended in 2–2.5 mL of buffer per gram of cells. The resuspension buffer consisted of 50 mM MES pH 6.5 at 4 °C, 0.002% (w/v) phenylmethanesulfonyl fluoride (PMSF) and one cOmplete™ EDTA-free protease inhibitor cocktail (Roche) per 50 mL. After homogenizing the cells, the resuspension was passed through a Z-plus 2.2 kW cell disruptor (Constant Systems Limited) once at 15,000 and twice at 30,000 psi. Cell debris was removed by centrifugation at 31,900×*g* in an SLA-1500 rotor (Sorvall) for 1 h and the membrane fraction was collected by ultracentrifugation at 234,800×*g* in a Ti45 rotor (Beckman) for 2 h. Membranes were suspended in 50 mM MES pH 6.5 at 4 °C and flash frozen in liquid N_2_.

### Enzyme purifications

*P. denitrificans* membranes (200–600 mg) were diluted to a concentration of 9.5 mg mL^–1^ in buffer with a composition of 20 mM MES pH 6.5 at 4 °C, 100 mM NaCl, 5 mM CaCl_2_, 10% glycerol, 0.002% PMSF, and the cOmplete™ EDTA-free protease inhibitor cocktail. Membranes were solubilized for 30 min at a 3:1 detergent to protein ratio by addition of 2.85% DDM (Anatrace) to membranes continuously stirred at 4 °C. Non-solubilized material was removed by centrifugation (172,000×*g*, 45 min). Imidazole was added to the supernatant to a final concentration of 20 mM, then the supernatant was filtered through a 0.45 µm filter. The supernatant was loaded onto either a 2 or 5 mL HisTrap HP column (Cytiva) and washed with buffer A (20 mM MES pH 6.5 at 4 °C, 400 mM NaCl, 5 mM CaCl_2_, 10% glycerol, 0.1% DDM, 40–80 mM imidazole) before complex I was eluted with buffer B (buffer A plus 200 mM imidazole). Fractions from the single eluted peak were combined, concentrated to 1 mL, and loaded onto either a Superdex 200 increase 10/300 GL or Superdex 200 5/150 GL column (Cytiva) equilibrated in buffer C (20 mM MES pH 6.5 at 4 °C, 150 mM NaCl, 10 mM CaCl_2_, 10% glycerol, 0.05% DDM). Fractions from the first peak corresponding to complex I were collected and concentrated to 10–20 mg mL^–1^ before glycerol was added at 20% and the protein flash frozen in liquid N_2_.

ATP synthase of *E. coli*^71^ and AOX of *Trypanosoma brucei brucei*^61^ were purified as described previously.

### Membrane and soluble *Pd*-CI kinetic assays

All kinetic assays were carried out at 32 °C in a Molecular Devices SpectraMax 348 96-well plate reader in buffer containing 10 mM MES (pH 6.5 at 32 °C), 25 mM NaCl and 2 mM CaCl_2_. NADH:O_2_ oxidoreduction was measured at 340–380 nm (ε = 4.81 mM^–1^ cm^–1^) and NADH:APAD^+^ (3-acetylpyridine adenine dinucleotide) oxidoreduction was measured at 400–450 nm (ε = 3.16 mM^–1^ cm^–1^). For NADH:O_2_ measurements, typically 5–10 µg mL^–1^ of membranes were assayed with 200 µM NADH and 12.5 µg mL^–1^ alamethicin. Complex I was inhibited with 5 µM piericidin A. Detergent-solubilized complex I (0.5 µg mL^–1^) was typically assayed for NADH:DQ activity in buffer containing 200 µM NADH, 200 µM DQ, 0.15% asolectin, 0.15% CHAPS (3-[(3-cholamidopropyl)dimethylammonio]-1-propanesulfonate). The NADH:APAD^+^ activity of membranes and *Pd*-CI were assayed in buffer containing 100 µM NADH, 500 µM APAD^+^ and 1 µM piericidin A. For membranes, 12.5 µg mL^–1^ alamethicin was also added to the buffer and for *Pd*-CI, 0.15% asolectin and 0.15% CHAPS were included. dNADH was used for comparison of membrane activities for the wild-type and NDH-2 containing variants. H_2_O_2_ production from *Pd*-CI was measured at 557–620 nm (ε = 51.6 mM^–1^ cm^–1^) using the horseradish peroxidase dependent oxidation of Amplex red to resorufin^72^. The reaction contained 30 µM NADH, 2 units mL^–1^ horseradish peroxidase, 10 µM Amplex red and 10 units mL^–1^ superoxide dismutase (SOD).

### Reconstitution of complex I into liposomes

For a standard reconstitution, 5 mg of synthetic lipids (all from Avanti Polar Lipids as 25 mg mL^−1^ stocks in chloroform) at a ratio of 52:37:8:3 DOPG:DOPC:DOPE:TOCL (% (w/w)), mimicking the natural lipid composition of *P. denitrificans* membranes^65^, and 50 nmol ubiquinone Q_10_ (from a 6.8 mM stock in chloroform) were mixed in a 25 mL round bottomed flask. For optimization of the reconstitution protocol, the total and relative amounts of lipids were varied as described in the figure legends. The chloroform was removed by rotating the flask under a nitrogen stream, then further for at least 1 h under vacuum in a desiccator. Liposomes were formed by the addition of 1 mL reconstitution buffer (10 mM MES, pH 6.5, 50 mM KCl, 250 mM sucrose), to achieve 5 mg lipid mL^−1^, and by vigorous mixing (Vortex Genie 2, Scientific Instruments) followed by 11 extrusions through a 100 nm Nucleopore polycarbonate membrane (Whatman). For reconstitution of *Pd*-CI, 200 µL liposomes (5 mg lipid mL^−1^) were partially solubilized by addition of 6 μL 20% (w/v) sodium cholate (0.5% final concentration) and the solution was incubated for 10 min on ice after mixing briefly by inversion. Then, 100 µg purified *Pd*-CI (13.4 mg mL^−1^) (and 50 µg *E. coli* ATP synthase for co-reconstitutions) and reconstitution buffer were added to yield a final volume of 250 μL. The solution was mixed by inversion, incubated on ice for 15 min, and cholate was removed using a PD10 desalting column (Cytiva) at 4 °C. PLs were collected by centrifugation at 100,000×*g* (4 °C for 45 min), resuspended in 100 μL ice-cold reconstitution buffer, and stored on ice until required.

### Characterization and kinetic assays of proteoliposomes

The amount of *Pd*-CI in PLs was calculated using a flavin-site NADH:APAD^+^ oxidoreduction assay^73^. The specific NADH:APAD^+^ activity of purified *Pd*-CI was measured in reconstitution buffer containing 1 µg mL^–1^ *Pd*-CI, 100 µM NADH, 500 µM APAD^+^, 1 µM piericidin A and 0.2% (w/v) DDM. To calculate the amount of outward facing *Pd*-CI in PLs, the specific activity of purified *Pd*-CI was compared to the activity of PLs in reconstitution buffer containing 100 µM NADH, 500 µM APAD^+^ and 1 µM piericidin A. Orientation and retention of *Pd*-CI were determined by adding 20 µg mL^–1^ alamethicin^61^ or 0.2% DDM, respectively, to the assay mixture. The total phospholipid and Q_10_ contents were determined as described previously^61,63^. Rates of NADH:O_2_ oxidoreduction were measured with 0.5 μg outward-facing CI mL^−1^ in the reconstitution buffer containing 200 μM NADH and 20 μg mL^−1^ AOX. For determination of the respiratory control ratio, an additional 10 µg mL^-1^ gramicidin A were used in the assay buffer^61^. Fluorescence quench assays were conducted in a Shimadzu RF-5301 PC spectrofluorometer as described previously^63^ using reconstitution buffer containing 1 μg outward facing CI mL^−1^, 20 μg AOX mL^−1^, 500 nM ACMA and 5 µM valinomycin. Fluorescence quenches were initiated by addition of 1 mM NADH and the ΔpH formed was released by the addition of 25 μg mL^−1^ alamethicin. ATP synthesis was measured at ambient temperature for 1 μg outward-facing CI mL^−1^ as described^63^.

### EPR spectroscopy

EPR samples were prepared anaerobically by transferring 100 µL of purified complex I (5.8 mg mL^–1^) to a 4 mm (O.D.) EPR tube (Wilmad), then the complex I was reduced by the addition of 15 mM NADH. The sample was immediately frozen in dry-ice/acetone before transfer to liquid nitrogen for storage. EPR measurements were performed using an X/Q-band Bruker Elexsys E580 Spectrometer (Bruker BioSpin) operating in X-band mode and equipped with a closed-cycle cryostat (Cryogenic Ltd) and X-band split-ring resonator module (ER 4118X-MD5). EPR measurement conditions were 100 kHz modulation frequency, 7 G modulation amplitude, 2 mW microwave power; other relevant measurement conditions are given in the figure legend. All spectra presented are baseline corrected using a buffer-only sample. EPR simulations were performed using the EasySpin package for MATLAB^74^.

### SDS-PAGE and mass spectrometry

Purified complex I was incubated in loading buffer (0.125 M Tris–HCl (pH 6.8), 20% (w/v) glycerol, 4% (w/v) SDS, 0.005% (w/v) bromophenol blue and 0.1 M DTT) for 10 min at room temperature and 10 µg loaded on a Novex WedgeWell 10–20% tris–glycine gel. The gel was run as described previously and bands visualized using Coomassie R250^75^. Bands excised from SDS-PAGE gels were digested with trypsin and analyzed by matrix-assisted laser-desorption ionization (MALDI) using an Applied Biosystems spectrometer. Spectra were assigned to peptide sequences and their originating proteins using the Mascot 2.4 application (Matrix Science Ltd.) with a peptide precursor mass tolerance of 360 ppm and fragment mass tolerance of 0.8 Da. One missed cleavage, plus methionine oxidation and cysteine propionamide formation as variable modifications were allowed.

## Supplementary Information

Supplementary Information.

## Data Availability

All data generated or analyzed during this study are included in this published article (and its Supplementary Information files) and/or are available from the corresponding author on reasonable request.
