# Supplementary material for: Paracoccus denitrificans: a genetically tractable model system for studying respiratory complex I
Source: Sci Rep. 2021 May 12;11:10143. doi: 10.1038/s41598-021-89575-9 (PMC8115037; doi:10.1038/s41598-021-89575-9)
Supplement: Supplementary file 1 — Supplementary Information. [file 41598_2021_89575_MOESM1_ESM.pdf]

Supplementary Information

***Paracoccus denitrificans*: a genetically tractable model system for studying respiratory complex I**

Owen D. Jarman, Olivier Biner, John J. Wright and Judy Hirst*

The Medical Research Council Mitochondrial Biology Unit, University of Cambridge, The Keith Peters Building, Cambridge Biomedical Campus, Hills Road, Cambridge CB2 0XY, UK

*Corresponding author

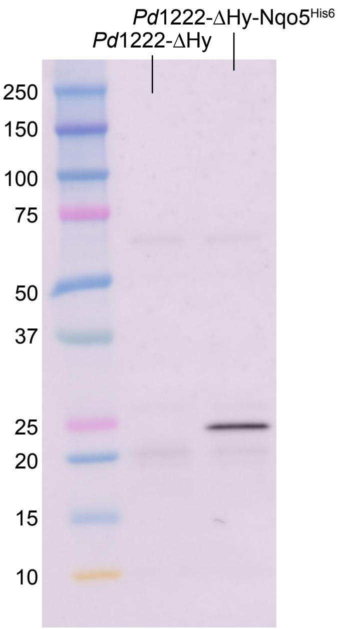

Figure S1. Western blot of SBPs from the *Pd*1222-ΔHy and *Pd*1222-ΔHy-Nqo5^{His6} strains. SBPs were prepared as described by Jarman et al.[41]. SBPs were incubated in loading buffer (0.125 M Tris-HCl (pH 6.8), 20% (w/v) glycerol, 4% (w/v) SDS, 0.005% (w/v) bromophenol blue and 0.1 M DTT) for 10 min at 70 ˚C. Each sample (20 µg) was loaded into a 10-20% Tris-Glycine Wedge well and 220 V applied for 50 min. Proteins were transferred onto a Immobilon-P PVDF membrane using transfer buffer containing 25 mM Tris, 0.192 M glycine, 20% (v/v) methanol at 4 ˚C and application of 300 mA for 1 hour. The membrane was then blocked with blocking buffer (1xTris-buffered saline (TBS), 5% (w/v) milk) for 1 hour and incubated overnight at 4 ˚C with 1:10000 dilution of His-tag anti-rabbit antibody. The membrane was then washed in 1xTBS, 0.1% (v/v) Tween-20 for 1 hour followed by incubation with the anti-rabbit IgG HRP Conjugate antibody in blocking buffer for 1 hour. The membrane was again washed in 1xTBS, 0.1% (v/v) Tween-20 for 1 hour followed by a final wash in 1xTBS. Chemiluminescence was detected after addition of ECL-Prime reagent (Amersham) and imaging on an Amersham imager 680. The full-length blot is shown. The original image is shown in figure S6.

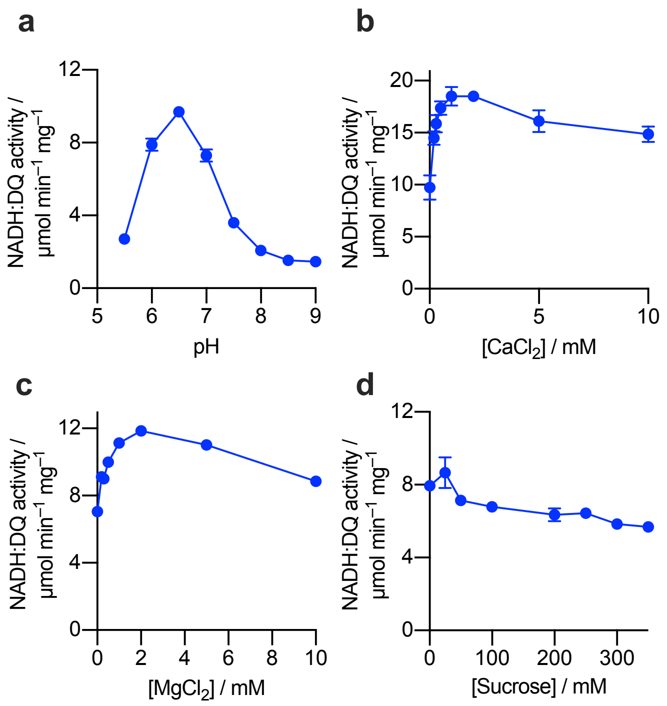

Figure S2. Optimization of the assay buffer for studying purified *P. denitrificans* complex I. Data points show average NADH:DQ activity for three technical replicates ± S.E.M.. (a) pH dependence of complex I. (b) $CaCl_2$ titration in pH 6.5 buffer. (c) $MgCl_2$ titration in pH 6.5 buffer. (d) Sucrose titration in pH 6.5 buffer.

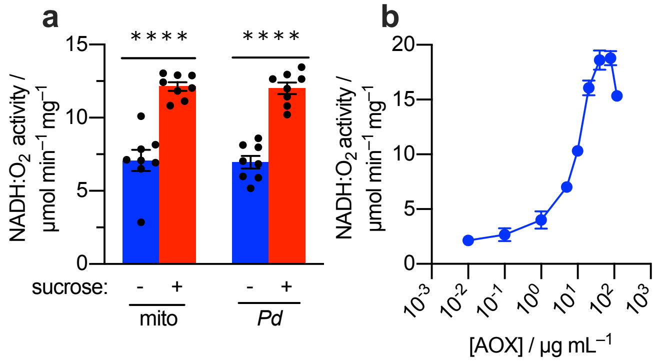

Figure S3. Optimization of *Pd*-CI-containing proteoliposomes. (a) 50 µg *Pd*-CI were reconstituted into 200 µL of liposomes at 5 mg mL^{-1} according to the standard protocol described in Materials and Methods. Reconstitutions were performed into liposomes containing 10 nmol Q_{10} (mg lipids)$^{-1}$ and phospholipid mixtures either mimicking the membrane composition of the inner mitochondrial membrane[64] (mito; 8:1:1 DOPC:DOPE:TOCL) or the cytoplasmic membrane of *P. denitrificans*[65] (*Pd*; 52:37:8:3 DOPG:DOPC:DOPE:TOCL). Reconstitutions into liposomes of both lipid compositions were carried out in 10 mM MES, pH 6.5 at 4 °C, 50 mM KCl in the presence or absence of 250 mM sucrose. Two individuals reconstitutions were performed and NADH:O_2 assays were measured as technical replicates (n = 4). Error bars show propagated errors (± S.E.M.) calculated from all measurements (n = 8). Statistical significance was calculated by one-way ANOVA using Tukey's test, **** = $p < 0.0001$. (b) The amount of AOX (in µg mL^{-1}: 0.01, 0.1, 1, 5, 10, 20, 40, 80, 160) added to the assay solution containing 0.5 µg mL^{-1} outward facing *Pd*-CI PLs was varied. Data are shown as mean averages with propagated errors (± S.E.M.) from technical replicates (n = 3). NADH:O_2 activities were measured as described in Materials and Methods.

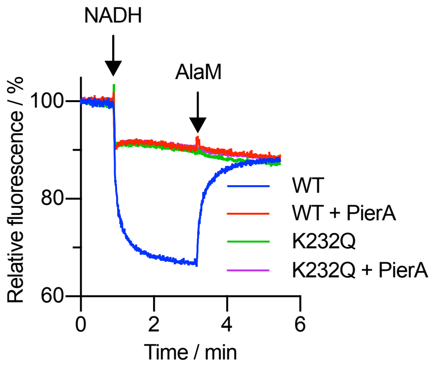

Figure S4. ACMA quenching in wild-type and K232Q^{Nqo13} complex I-containing proteoliposomes. Proton pumping was initiated by addition of 1 mM NADH and ΔpH dissipated by addition of 25 µg mL^{-1} alamethicin (AlaM). Piericidin A (5 µM) was used to inhibit complex I before NADH addition.

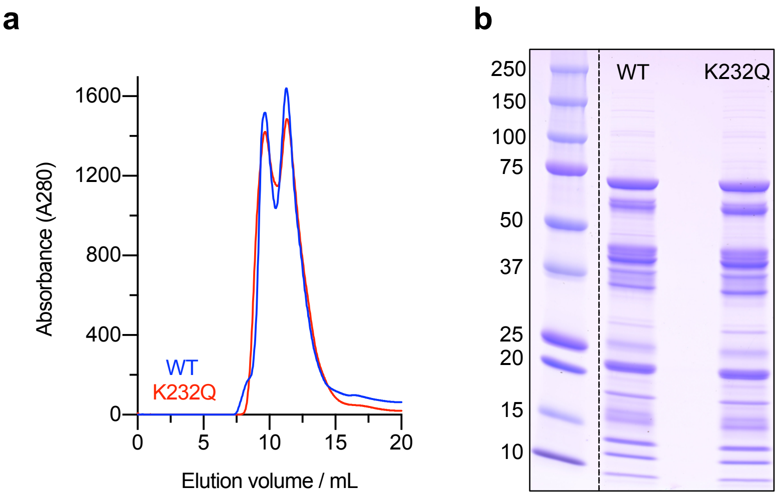

Figure S5. Gel filtration trace and SDS-PAGE of purified wild-type and the K232Q^{Nqo13} mutant. (a) The wild-type and K232Q^{Nqo13} mutants were purified on a Superdex 200 increase 10/300 GL column. For comparison, the elution traces were aligned to the peak absorbance for the complex IV elution (the second peak), which should remain unchanged between complex I variants. (b) The wild-type and K232Q^{Nqo13} mutants were analyzed by SDS-PAGE. The band/subunit pattern for both were the same. Three full-length lanes are shown from a single gel; the image has been cut and they have been moved to be adjacent to each other. The original image is shown in figure S6.

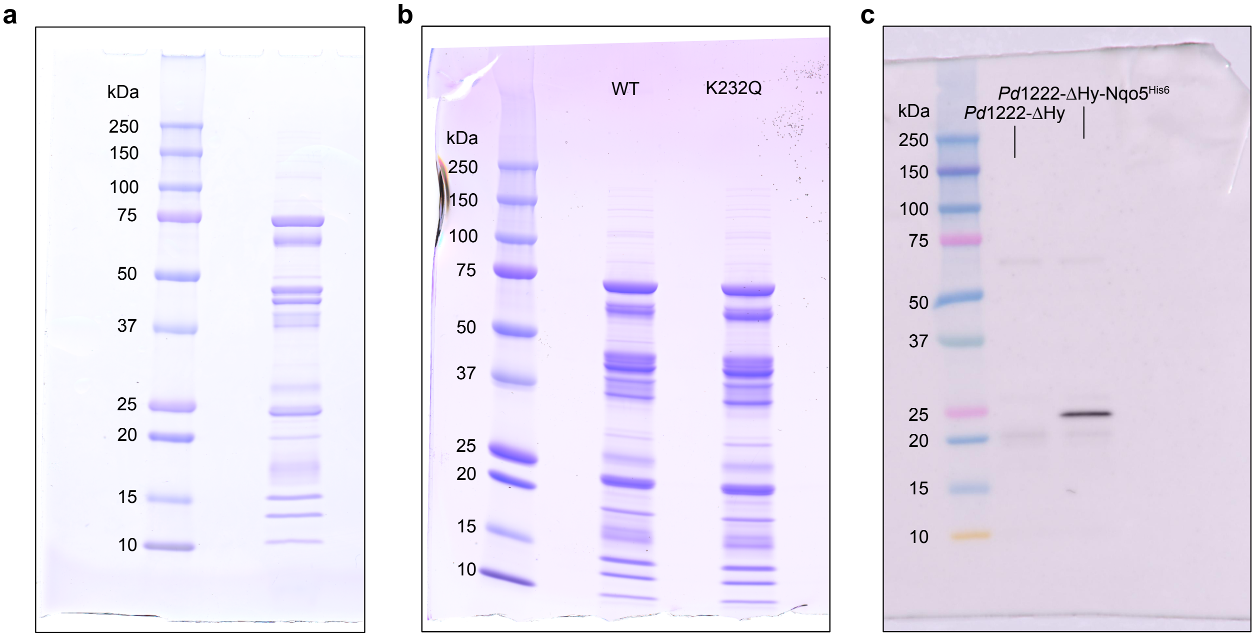

Figure S6. Original gel and blot images. (a) Full-length, unprocessed SDS-PAGE gel as shown in figure 1b of the manuscript. (b) Full-length, unprocessed SDS-PAGE gel of wild-type and K232Q^{Nqo13} mutants as shown in figure S5. (c) Full-length, unprocessed Western blot of *Pd*1222-ΔHy and *Pd*1222-ΔHy-Nqo5^{His6} as shown in figure S1.

Table S1. Complex I subunits and proteins identified in purified *P. denitrificans* complex I. Complex I constituents were resolved by SDS-PAGE and protein bands were excised. Protein bands were digested with trypsin and peptides were identified by MALDI-TOF/TOF mass spectrometry and assigned to a subunit/protein by peptide mass fingerprinting. The number of unique peptides identified with scores above the Mascot 95% confidence limit are shown along with the protein score and total Mascot MS/MS Ions score for the protein.

		Peptide mass fingerprinting			Tandem MS	
**Subunit/protein**	**PRIDE/Pden number**	**Mascot score**	**Peptides (n)**	**% coverage**	**Peptides (n)**	**Mascot MS/MS score**
Nqo1	A1B491/Pden_2243	228/50	9	19	3	182/21
Nqo2	A1B494/Pden_2246	368/50	5	27	4	278/22
Nqo3	A1B489/Pden_2241	657/50	13	16	7	453/21
Nqo4	A1B495/Pden_2247	244/50	5	14	3	225/21
Nqo5	A1B496/Pden_2248	124/50	2	17	1	110/22
Nqo6	A1B497/Pden_2249	337/50	6	29	5	210/21
Nqo7	A1B498/Pden_2250	n. d.	n. d.	n. d.	n. d.	n. d.
Nqo8	A1B487/Pden_2239	56/50	4	4	1	21/21
Nqo9	A1B486/Pden_2238	483/50	9	37	7	286/21
Nqo10	A1B483/Pden_2235	292/50	4	32	3	203/21
Nqo11	A1B482/Pden_2234	n. d.	n. d.	n. d.	n. d.	n. d.
Nqo12	A1B481/Pden_2233	646/50	8	18	8	473/21
Nqo13	A1B480/Pden_2232	112/50	4	7	2	82/21
Nqo14	A1B479/Pden_2231	240/50	4	14	4	167/21
PdN7BM	A1B1H8/Pden_1267	513/50	4	63	4	420/21
PdNUYM	A1B1M0/Pden_1309	436/50	8	70	5	318/21
PdNUMM	A1B357/Pden_1854	149/50	4	56	2	74/21
Transcription termination factor Rho	A1AXX5/Pden_0002	257/50	2	10	2	233/21
Protein-L-isoaspartate O-methyltransferase	A1B5L6/Pden_2726	152/50	2	20	2	129/21
Elongation factor Tu	A1B002/Pden_0734	105/50	1	4	1	105/21
Glutamine–fructose-6-phosphate aminotransferase	A1AZN7/Pden_0619	50/50	3	2	1	44/21
cytochrome b	A1B4F3/Pden_2306	35/50	1	4	1	35/21
Ubiquinol-cytochrome c reductase iron-sulfur subunit	A1B4F2/Pden_2305	43/50	2	4	1	30/21
Type I secretion target repeat protein	A1B5Q1/Pden_2761	28/50	1	2	1	28/21
Sulfate adenylyltransferase subunit	A1BAB5/Pden_4395	22/50	1	5	1	22/20
Uncharacterized protein	A1AY62/Pden_0089	22/50	1	2	1	21/21

Table S2. EPR simulation parameters for the *Pd*-CI FeS clusters.

Cluster	g_x	g_y	g_z
**N1b**	1.936	1.939	2.021
**N2**	1.923	1.924	2.055
**N3**	1.867	1.924	2.039
**N4**	1.884	1.939	2.103

Table S3. N-ethylmaleimide (NEM) sensitivity of *P. denitrificans* membranes. Membranes (5 mg mL^{-1}) were either incubated on ice for 30 min or 'deactivated' by incubation at 37 ˚C for 30 min in the presence of a cOmplete™ EDTA-free protease inhibitor cocktail. Membranes were then diluted to 2 mg mL^{-1} and incubated with 2 mM NEM or DMSO (control) for 20 min on ice. The NADH:O_2 oxidoreduction activities of the membranes were then measured. There was no significant decrease in activity in NEM-treated membranes in either the control or 'deactivated' samples. Activities are reported as average ± S.E.M. (n = 3 technical replicates).

Pre-treatment	NADH:O_2 activity / μmol min^{-1} mg^{-1}	
	+ NEM	+ DMSO
As-is	0.854 ± 0.011	0.812 ± 0.032
'Deactivated'	0.800 ± 0.013	0.817 ± 0.025
